# Over Expression of Wild Type or a Catalytically Dead Mutant of SIRTUIN 6 Does Not Influence NFκB Responses

**DOI:** 10.1371/journal.pone.0039847

**Published:** 2012-07-06

**Authors:** Rachel Grimley, Oxana Polyakova, Jessica Vamathevan, Joanne McKenary, Brian Hayes, Champa Patel, Janet Smith, Angela Bridges, Andrew Fosberry, Anshu Bhardwaja, Bernadette Mouzon, Chun-Wa Chung, Nathalie Barrett, Nicola Richmond, Sundip Modha, Roberto Solari

**Affiliations:** 1 Platform Technology Sciences, GlaxoSmithKline, Gunnels Wood Road, Stevenage, Hertfordshire, United Kingdom; 2 Computational Biology, GlaxoSmithKline, Gunnels Wood Road, Stevenage, Hertfordshire, United Kingdom; 3 Allergic Inflammation Discovery Performance Unit, GlaxoSmithKline, Gunnels Wood Road, Stevenage, Hertfordshire, United Kingdom; Chinese University of Hong Kong, Hong Kong

## Abstract

SIRT6 is involved in inflammation, aging and metabolism potentially by modulating the functions of both NFκB and HIF1α. Since it is possible to make small molecule activators and inhibitors of Sirtuins we wished to establish biochemical and cellular assays both to assist in drug discovery efforts and to validate whether SIRT6 represents a valid drug target for these indications. We confirmed in cellular assays that SIRT6 can deacetylate acetylated-histone H3 lysine 9 (H3K9Ac), however this deacetylase activity is unusually low in biochemical assays. In an effort to develop alternative assay formats we observed that SIRT6 overexpression had no influence on TNFα induced nuclear translocation of NFκB, nor did it have an effect on nuclear mobility of RelA/p65. In an effort to identify a gene expression profile that could be used to identify a SIRT6 readout we conducted genome-wide expression studies. We observed that overexpression of SIRT6 had little influence on NFκB-dependent genes, but overexpression of the catalytically inactive mutant affected gene expression in developmental pathways.

## Introduction

Sirtuins are a family of NAD^+^ dependent enzymes that are conserved from prokaryotes to man and the founding member of the family, Sir2 (silent information regulator protein), was discovered in yeast as a protein that could control longevity. In mammals there are 7 members of the Sirtuin family, SIRT1-7, and they have been linked to many biological processes ranging from chromatin modification, genomic stability, metabolism, cellular senescence and organismal lifespan. The Sirtuins are known to catalyse two different NAD^+^ dependent post-translation protein modification reactions, namely deacetylation and ADP-ribosylation. During the protein deacetylation reaction, NAD^+^ is consumed as a co-substrate and cleaved to nicotinamide and 2′-O-acetyl-ADP-ribose. All Sirtuins, except SIRT4, display NAD^+^ dependent deacetylase activity and SIRT4 and SIRT6 have been shown to display additional ADP-ribosyl transferase activity [1].

SIRT6 and SIRT7 cDNAs were originally discovered by searching an EST database with SIRT4 as the probe [2] and SIRT6 mRNA and protein are expressed in most mouse tissues examined, with the highest protein levels in thymus, skeletal muscle, and brain [3]–[5]. Subcellular fractionation studies showed that SIRT6 is predominantly localized to the chromatin/nuclear matrix fractions and immunocytochemistry studies on transfected cells confirmed its nuclear localisation [3]–[5]. SIRT6 knockout mice display a greatly shortened lifespan and acute degenerative and metabolic defects similar to premature aging pathologies. It was further shown that SIRT6 knockout embryonic stem cells and mouse embryonic fibroblasts showed impaired proliferation and increased sensitivity to DNA-damaging agents [5]. These studies demonstrated that SIRT6 promotes resistance to DNA damage and suppresses genomic instability, consistent with a role in base excision repair (BER) although double-strand break (DSB) repair and cell cycle checkpoints appeared normal [5]. Recent studies have gone on to show that SIRT6 is involved in DSB repair by forming a macromolecular complex with DNA-dependent protein kinase [6] and SIRT6 promotes DNA end resection through CtIP acetylation [7]. More recently SIRT6 has been shown to promote DNA repair by ADP-ribosylating and activation of PARP1 [8].

**Figure 1:**
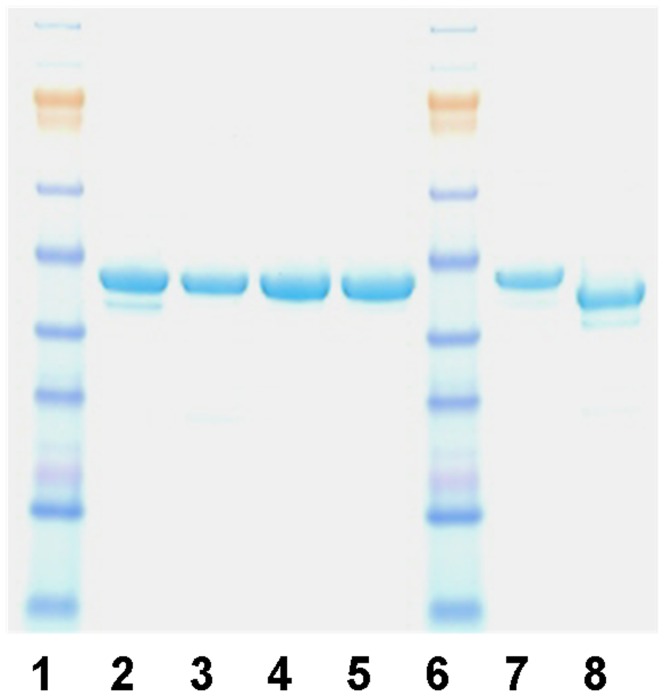
Full length SIRT6 and mutant expression. Human SIRT6 was amplified by PCR and cloned into a modified pET28a expression vector which incorporated a FLAG tag upstream of a 6xHIS tag and thrombin cleavage site (th). In addition to this Flag-6His-th-tag, the N-terminus of SIRT6 was modified with a Tev or Precission protease site. Wild type SIRT6 and a series of site directed mutants were expressed and purified as described. Samples were analysed by SDS-PAGE. Lanes 1 and 6: Molecular mass markers; Lane 2: wild type SIRT6; Lane 3: H133W SIRT6; Lane 4: H133Y SIRT6; Lane 5: N114A SIRT6; Lane 7: wild type SIRT6 with Precission tag; Lane 8: wild type SIRT6 with N-terminal tag removed.

Few direct substrates for SIRT6 have been described so far but it has been shown that the human SIRT6 protein is an NAD^+^-dependent histone H3 lysine-9 (H3K9) deacetylase that modulates telomeric chromatin. SIRT6 associates specifically with telomeres, and SIRT6 depletion leads to telomere dysfunction with end-to-end chromosomal fusions and premature cellular senescence. Moreover, SIRT6-depleted cells exhibited abnormal telomere structures that resemble defects observed in Werner syndrome, a premature aging disorder [9]. At telomeric chromatin, SIRT6 deacetylated H3K9 and was required for the stable association of RECQL2/WRN, the factor that is mutated in Werner syndrome. Consequently SIRT6 contributes to the propagation of a specialized chromatin state at telomeres, which in turn is required for proper telomere metabolism and function. These findings constituted the first identification of a physiologic enzymatic activity of SIRT6, and linked chromatin regulation by SIRT6 to telomere maintenance and a human premature aging syndrome.

**Figure 2:**
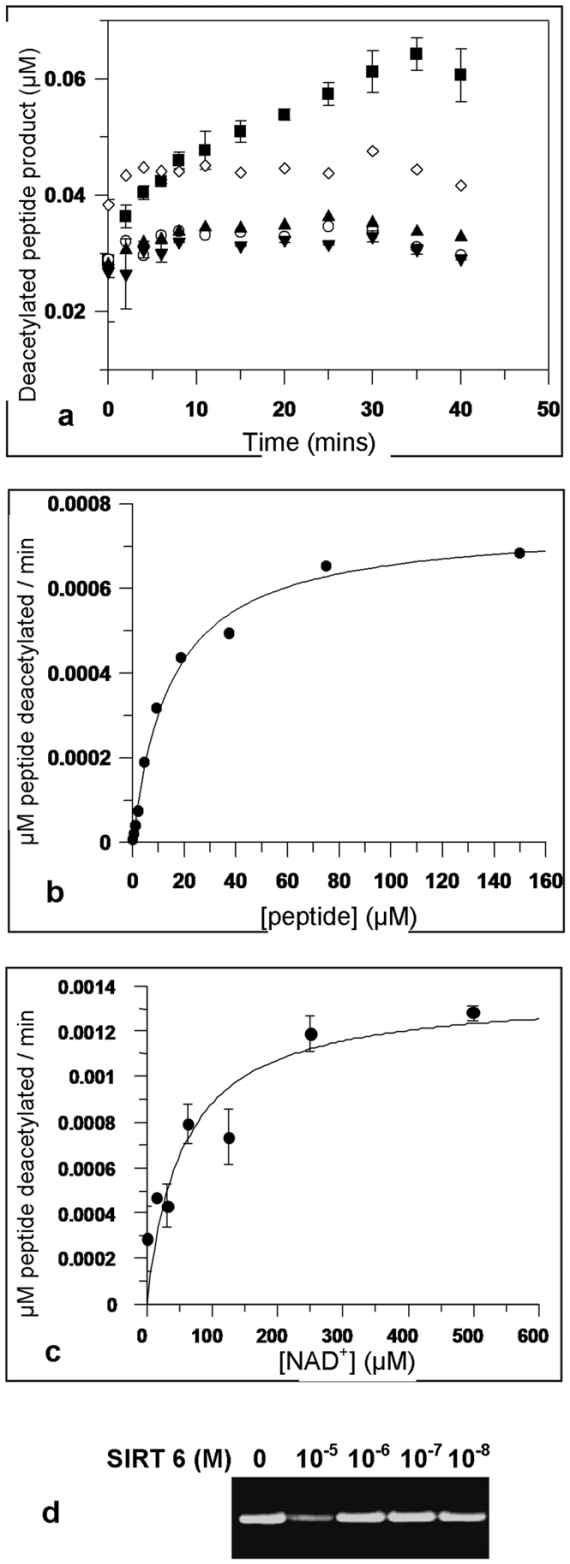
Kinetic characteristics of SIRT6 deacetylase activity. (**A**) Progress curves for purified, recombinant full-length wild-type (wt) and mutant SIRT6 proteins, expressed in *E.coli*. ▪ wt SIRT6 (2 µM), ▾H133W (10 µM), ▴ H133Y (10 µM), ○ N114A (10 µM), ◊ inhibition of wt (2 µM) with 20 mM NAM (this also generates an intrinsic level of background fluorescence). Error bars represent the standard error of mean duplicate values. (**B**) *K* ***_m,app_*** determination for peptide gave a value of 14.7+/−1.5 µM. Error bars represent the standard error of mean duplicate values. (**C**) *K* ***_m,app_*** determination for NAD^+^ gave a value of 52.4+/−18 µM. Error bars represent the standard error of mean duplicate values. (**D**) Deacetylation of purified H3/H4 histones with SIRT6. Deacetylation could be detected at a substrate:enzyme ratio of 1∶1.

An additional function for SIRT6 has recently been discovered, as a transcriptional regulator through its physical interaction with the transcription factors NFκB [10] and HIF1α [11]. Activation of RelA/p65 is proposed to recruit SIRT6 to the chromatin of NFκB target genes where it deacetylates acetylated histone H3 lysine-9 (H3K9Ac), leading to condensation of chromatin and thus terminating NFκB signalling [10]. In SIRT6 deficient cells H3K9 was hyperacetylated at NFκB sites within promoters leading to increased inflammatory gene expression, cellular senescence and apoptosis [10].

**Figure 3:**
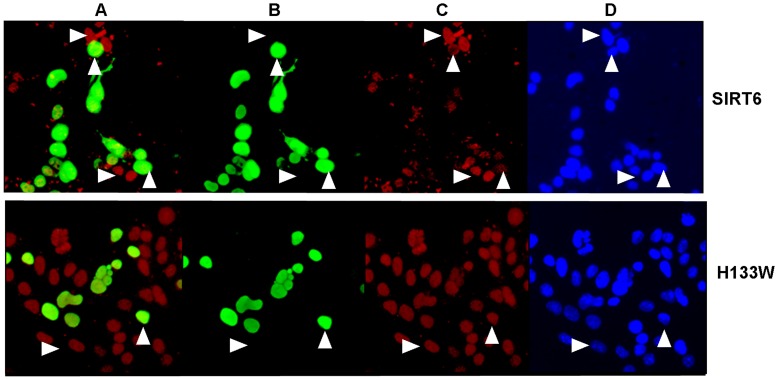
SIRT6 activity in mammalian cells. Panel A shows SIRT6 (anti-Flag stained in green) and H3K9Ac (red) double staining. Panel B shows SIRT6 staining alone. Panel C shows H3K9Ac staining alone and panel D shows Hoechst staining. Top row shows wild type SIRT6 transfection and lower row shows H133W transfection. Upward pointing arrowheads show the position of a transfected cell nucleus whereas a right pointing arrowhead shows an untransfected cell nucleus.

Chronic obstructive pulmonary disease (COPD) is a major global health problem characterised by chronic airflow limitation. There is good clinical evidence to suggest that the lungs of COPD patients have features of increased inflammation and accelerated aging [12]. The accelerated aging is evident both from the gross anatomical and functional changes that characterise emphysema and at a cellular level there is evidence for increased cellular senescence and telomere shortening thought to result from DNA damage or impaired DNA repair as a result of oxidative stress from cigarette smoke. Given the biology known about SIRT6 we wished to develop screening assays to discover activators or inhibitors of SIRT6 and evaluate its potential as a therapeutic target for COPD.

## Results

### Kinetic Characteristics of SIRT6 Deacetylase Activity

We quantified the deacetylation activity of SIRT6 using a peptide based around the sequence of H3K9Ac as a substrate. Lysine deacetylation activity has been demonstrated for human SIRT6 using H3K9Ac peptides [9] and H3K56Ac peptides and *in vivo* [13], [14] using HPLC and Western blot methods; however, we describe a detailed characterisation of basic kinetic parameters (*k_cat_, K_m,app_*_(peptide, NAD)_) using a modification of the quenched-Fluorescence Resonance Energy Transfer (FRET) method previously described for SIRT1 [15].

**Figure 4:**
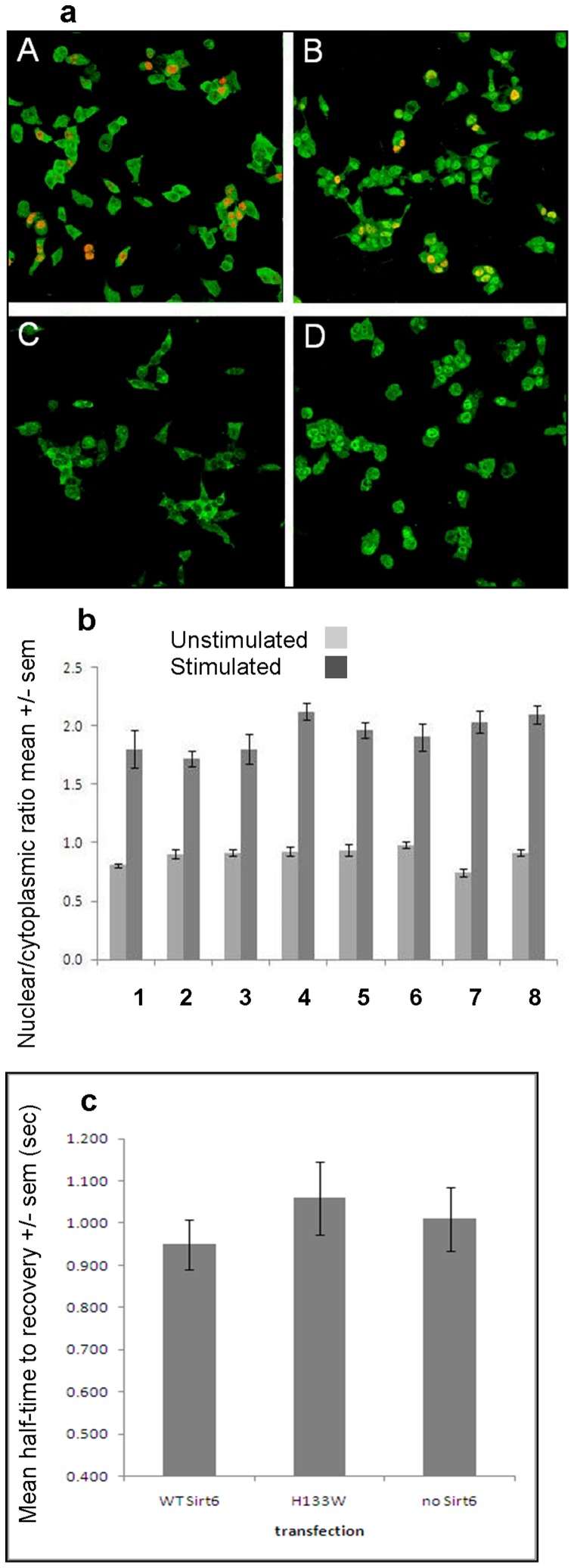
Influence of SIRT6 on nuclear translocation and nuclear mobility of RelA/p65. (**a**) Medium magnification confocal microscopy images of cells immunostained for RelA/p65 (green) and flag tag-SIRT6 (red). The images show cells with low intensity of RelA/p65 staining in the nucleus in unstimulated cells (A and C) and with high intensity for RelA/p65 in the nucleus after stimulation with TNFα (B and D). A and B are cells that had been transfected with 5 µg of flag-tagged wild-type SIRT6 and C and D cells which had a control transfection with an empty vector. Transfected cells that were stimulated by TNFα show orange/yellow nuclei demonstrating co-localisation of immunofluorescence for RelA/p65 and flag tag. **Figure 4b** Histograms of measurements of RelA/p65 nuclear:cytoplasmic ratio. The data shows an approximate doubling of this ratio after stimulation by TNFα. There were no significant differences between the responses of of untransfected cells (column 8), cells transfected with an empty vector (column 7) or cells transfected with various concentrations of wild-type (WT) SIRT6 vector (column 1, 0.5 µg; column 2, 2 µg: column 3, 5 µg) or H133W mutant SIRT6 vector (column 4, 0.5 µg; column 5, 2 µg; column 6, 5 µg). The ratios are the mean results for 100 cells (+/− SEM). **Figure 4c** Histogram showing the intranuclear mobility of YFP-RelA/p65 measured as half-time to recovery after photo-bleaching. Mean data from 40 cells measured in 2 experiments. YFP-RelA/p65 was not significantly more mobile in cells following transient transfection and overexpression of wild type SIRT6 than in cells following a control transfection of H133W or empty plasmid.

For this study we expressed full-length human SIRT6 in *E.coli* with either an N-terminal Flag-His-tag or with no tag following removal of the tag by Tev protease treatment. We also produced a number of site directed mutants of SIRT6. Histidine 133 (H133) is universally conserved amongst Sirtuins and recognises 3′OH group of NAD^+^. It is important for substrate binding and activation of the 2′OH group for nucleophilic attack on the peptidylimidate intermediate which activates the downstream catalytic events [16]. We made H133Y and H133W mutants, both of which expressed well in *E coli,* were purified and showed identical circular dichroism (CD) spectra to the wild type enzyme (data not shown). Amino-acids N114 and D116 are also conserved amongst Sirtuins, and are located within the NAD^+^ binding pocket. In order to examine these residues, we also made the N114A mutant which was purified and showed identical CD spectra to the wild type enzyme; however the D116A mutant expressed poorly and had evidence of misfolding. The various purified SIRT6 proteins used in this study are shown (Figure 1). For comparison we also assessed the enzymatic activity of immuno-precipitated Flag-tagged SIRT6 from transiently transfected HEK293 cells.

Our quantitative assays revealed a very low *k_cat_* value (0.001 min^−1^+/−0.00006, representing the overall first-order rate of the reaction), (Figure 2a) which is similar to previous qualitative assays [9] and slightly lower than the value described in a thorough quantitative biochemical analysis which reported a *k_cat_* of 0.00022 s^−1^ [17]. Literature for SIRT1, 2 & 3 report *k_cat_* values in the order of 12 min^−1^, and 0.32s^−1^ for the yeast Hst2 enzyme [18] hence the value we obtained for SIRT6 is several orders of magnitude lower. The apparent *K_m_* values for the peptide substrate (∼14+/−1.5 µM) and NAD^+^ (52+/−18 µM) are reasonably in line with values derived for other Sirtuins characterised in a similar manner (Figure 2b and 2c) [18] and correspond well with recent data generated for SIRT6 which reported a K_d_ for NAD^+^ of 27 µM [17]. Interestingly SIRT6 immuno-precipitated from HEK293 cells showed similar low levels of deacetylase activity using the H3K9Ac peptide substrate (*k_cat_  = *0.001 min^−1^+/−0.00001), as did the tagged and de-tagged recombinant SIRT6; hence we are confident of the overall integrity of the protein in terms of lack of interference from the N-terminal label [19]. As anticipated, the H133W/Y, and N114A mutants were, effectively, inactive (Figure 2a).

We were unable to quantify the reaction using purified H3/H4 histones as substrate due to the signal being lower than the detection limit of a ^14^C-NAD^+^ release assay [20]. However using Western blotting we were able to detect a low level of H3K9 deacetylation of purified H3/H4 histones at high ratios of enzyme to substrate (Figure 2d). In this assay the histone substrate was present at 10 µM and deacetylation could only be detected after a 2 hour incubation when SIRT6 was also present at 10 µM.

### Confirmation of SIRT6 Deacetylase Activity in Mammalian Cells

Having demonstrated a surprisingly low *k_cat_* value for SIRT6 deacetylase activity in biochemical assays, we wished to confirm its ability to deacetylate H3K9Ac in cellular systems prior to examining the functional influence of SIRT6 over expression on NFκB dependent gene expression. Furthermore, we wished to develop a high throughput and high content cell-based assay for SIRT6 using a cell imaging technique. In cells over expressing wild type SIRT6 there was a clear reduction in the level of nuclear H3K9Ac levels compared to untransfected cells in the same field (Figure 3), however, there was no such effect seen with H133W (or with H133Y or N114A mutants - data not shown). Consequently we believe that under these conditions SIRT6 can behave as a functional H3K9Ac deacetylase in cells and that the H133W and H133Y mutants are effectively “deacetylase dead”. Moreover, these studies established conditions of SIRT6 transfection and over expression that clearly decreased levels of H3K9 acetylation for subsequent functional experiments.

### Effect of SIRT6 Over Expression on Nuclear Trafficking of NFκB

Reduction of SIRT6 levels in cells has been associated with an enhanced activity of NFκB resulting in an increased expression of target genes and occupancy of RelA/p65 at NFκB responsive promoters [10]. Since NFκB dynamically traffics in and out of the nucleus following stimulation with TNFα we reasoned that any factor that resulted in enhanced binding of NFκB to DNA would shift the equilibrium distribution and be seen as a change in the nuclear/cytoplasmic ratio of the transcription factor. We wished to explore if SIRT6 could affect the function of NFκB and whether this alteration could be revealed by changes in its cellular distribution in response to upstream signals. Furthermore, if such an alteration could be revealed whether such a readout could be used as an assay for SIRT6 activators or inhibitors. Consequently, we made use of a well established model whereby TNFα stimulation causes translocation of RelA/p65 from the cytoplasm to the nucleus which can be detected by confocal microscopy and quantified as a nuclear/cytoplasmic ratio. HEK293 cells were transfected with increasing doses of plasmid expressing wild type or H133W SIRT6, or transfected with an empty plasmid or simply left untransfected, followed by treatment with 10 ng/ml TNFα for one hour. Cells were then fixed and stained for RelA/p65 and the nuclear/cytoplasmic ratio determined by confocal microscopy and image analysis. In all cases in resting cells the nuclear/cytoplasmic ratio of RelA/p65 distribution was between 0.8 and 1.0 and this increased to 1.8–2.2 following addition of TNFα as a result of nuclear translocation and DNA binding. Neither over expression of wild type SIRT6 nor the H133W mutant had any significant effect on RelA/p65 nuclear translocation (Figure 4a and 4b). In all over expression studies the conditions we use have a transfection efficiency of between 20–50% and there is no obvious cytotoxicity.

We reasoned that if SIRT6 was altering chromatin in such a way as to repress NFκB binding, this might be detected by a change in nuclear mobility of the transcription factor. So to extend our previous study we went on to examine the mobility of RelA/p65 within the nucleus by making use of a Yellow Fluorescent Protein-p65 (YFP-p65) fusion and a recovery after photo-bleaching technique [21]. HEK293 cells were transiently transfected with a vector expressing a YFP-p65 chimera and cells with moderate nuclear expression were selected for imaging by confocal microscopy. Rectangular regions of single nuclei corresponding to about 25% of the nuclear area were selected and bleached. Two pre-bleached images were taken, followed by 2 bleached frames and imaging was continued for a further 30 seconds to record the recovery from photobleaching. The intensity of YFP fluorescence during recovery within the bleached area was monitored and fitted an exponential curve to the data. We were able to show that over expression of SIRT6 or the H133W catalytically dead mutant had no significant influence on the half-time to recovery after photobleaching of YFP-p65 suggesting that SIRT6 does not influence the nuclear mobility of RelA/p65 (Figure 4c).

### Influence of SIRT6 Over Expression on NFκB Dependent Gene Expression

**Figure 5:**
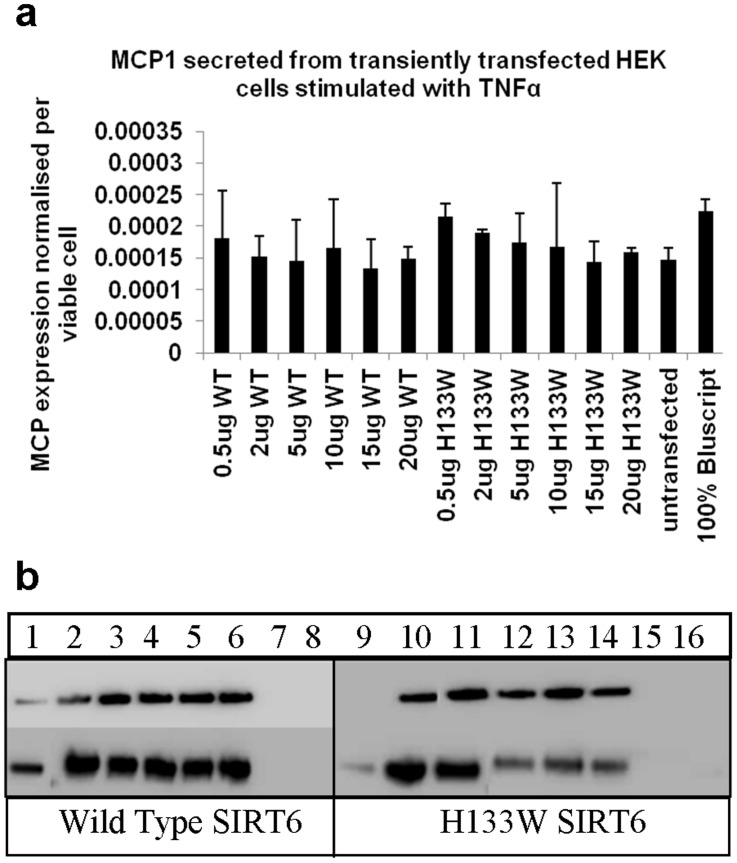
Influence of SIRT6 over expression on TNFα induced MCP1 expression. (**a**) HEK293 cells were transiently transfected with increasing amounts (0.5–20 µg) of expression plasmids for wild type SIRT6 or the H133W mutant. Control cells were transfected with an empty plasmid (100% Bluescript) or untransfected. Cells were stimulated with 10 ng/ml TNFα for 48 hours and MCP1 secretion into the culture supernatant was measured and normalised per live cell. Results represent means +/− SEM from triplicate experiments. (**b**) HEK293 cells were transiently transfected as above with increasing amounts (0.5–20 µg) of expression plasmids for wild type SIRT6 (lanes 1–6) or the H133W mutant (lanes 9–14). Control cells were transfected with an empty plasmid (lanes 7 and 15) or untransfected (lanes 8 and 16). Cells were stimulated with 10 ng/ml TNFα for 48 hours. Cell extracts were made and analysed by SDS-PAGE and Western blotting as described. Blots were probed with antibodies to FLAG (upper panel) and SIRT6 (lower panel).

Chromatin histone immunoprecipitation (ChIP) studies have demonstrated that following TNFα stimulation H3K9 acetylation is induced at the promoters of numerous NFκB target genes. Moreover, in cells depleted of SIRT6, H3K9 acetylation is increased resulting in enhanced gene expression [10]. Having established conditions where SIRT6 over expression in HEK293 cells reduced global levels of nuclear H3K9 acetylation as detected by confocal microscopy, we asked whether increased levels of SIRT6 could influence NFκB target gene expression. We therefore examined the effect of increasing SIRT6 levels on TNFα induced expression of MCP1, an inflammatory cytokine whose expression is known to be dependent on NFκB [22]. This was carried out by first measuring MCP1 protein levels secreted by HEK293 cells in response to TNFα stimulation. In this experiment, over expression of wild type SIRT6 or the H133W mutant had no effect on the level of MCP1 protein secreted in response to TNFα (Figure 5a). Cell extracts were also prepared for Western blotting with anti-Flag and anti-SIRT6 as transfection controls (Figure 5b) and in all cases experiments included an empty vector control and cell viability was monitored and MCP1 secretion was normalised per viable cell.

**Figure 6:**
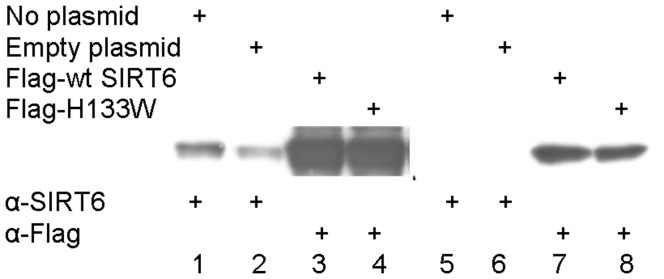
Over expression of SIRT6 or H133W. HEK293 cells were transfected with plasmid expressing Flag-tagged wild type SIRT6 (lanes 3 and 7) or deacetylase-dead (H133W) mutant of human SIRT6 in (lanes 4 and 8). Control cells were transfected with no DNA (lanes 1 and 5) or empty vector DNA pCDNA3 only (lanes 2 and 6). Each lane was loaded with 50 µg total protein of cell extract and analysed by SDS-PAGE followed by Western blotting with either anti-SIRT6 or anti-Flag. RNA was extracted from these cells was used for transcriptomic analysis without and with TNFα stimulation for 4 h.

We went on to examine the effects of SIRT6 on gene expression both to study the influence of SIRT6 on NFκB dependent signalling and to identify a signature of genes that are influenced by SIRT6 that could be used as a phenotypic readout for SIRT6 activity. We transfected HEK293 cells with expression vectors encoding wild type SIRT6 or the H133W mutant followed by stimulation with TNFα for 4 hours and purified total RNA for microarray analysis. Expression of the SIRT6 or H133W proteins were confirmed by Western blotting of cell extracts (Figure 6). Controls included untransfected cells and cells transfected with an empty vector prior to TNFα stimulation which resulted in 8 test conditions, each performed in triplicate.

Analysis of mRNA transcripts with a fold change (FC) restriction of 1.5 and False Discovery Rate (FDR) corrected *p* value of 0.05 was performed for a variety of comparisons (Table 1). The complete and detailed lists of significant genes from all comparisons are provided in File S1. Analysing the effect of TNFα stimulation on cells transfected with empty vector (comparison 1 Table 1) we identified 183 genes, which were induced, whereas the transcription of 102 genes was down-regulated. When analysing changes induced by TNFα in untransfected cells (comparison 2 Table 1), we found 194 transcripts upregulated and 60 downregulated. There was a high degree of overlap between comparison 1 and comparison 2 showing that transfection with empty vector had little influence on the TNFα response. This overlapping set of 153 upregulated genes and 45 downregulated genes was used in all subsequent comparisons and used as our reference “TNFα profile” of 198 transcripts. This “TNFα profile” showed 53% similarity to a profile obtained from A549 cells exposed to TNFα for 4 hours [23]. The “TNFα profile” showed significant enrichment for genes regulated by NFκB but also a number of other transcription factors (Table 2). Consequently we were confident that our conditions for stimulation of HEK293 cells with TNFα generated a robust and clear NFκB response.

**Table 1:** The effect of TNFα stimulation in the presence of SIRT6 and H133W transfects on genome-wide expression.

Comparison	Agent	Compared to	FC >1.5	FC<−1.5
1	h. empty vector + TNFα	g. empty vector	183 (240)	102 (112)
2	b. TNFα only	a. unstimulated	194 (262)	60 (70)
*Overlap between comparisons 1 and 2 (TNFα profile)*			153 (199)	45 (51)
3	c. SIRT6	g. empty vector	27 (27)	0 (0)
4	e. H133W	g. empty vector	244 (288)	8 (8)
5	d. SIRT6+ TNFα	h. empty vector + TNFα	38 (41)	2 (2)
6	f. H133W + TNFα	h. empty vector + TNFα	356 (419)	59 (59)

Gene expression comparisons and resulting numbers of significant genes (−1.5< FC <1.5; *p* value <0.05). Numbers in parentheses indicate the numbers of significant probesets in each comparison.

**Table 2:** Transcription factors (TF) significantly connected to 198 genes in the “TNFα profile”.

TranscriptionFactor	Actual^a^	R	Expected^b^	Ratio^c^	p-value^d^	z-score^e^
RelA (p65 NF-kBsubunit)	47	451	3.982	11.8	1.011E-36	21.87
c-Rel (NF-kBsubunit)	37	330	2.914	12.7	5.056E-30	20.2
NF-kB1 (p50)	29	200	1.766	16.42	6.663E-27	20.68
SP1	57	2410	21.28	2.679	1.873E-12	8.228
RelB (NF-kBsubunit)	10	41	0.362	27.62	2.030E-12	16.1
c-Jun	22	475	4.194	5.245	2.641E-10	8.826
CREB1	32	1024	9.042	3.539	4.662E-10	7.848
EGR1	24	587	5.183	4.631	4.826E-10	8.412
ETS1	19	462	4.079	4.658	3.155E-08	7.497
STAT3	17	373	3.293	5.162	3.969E-08	7.649
NF-kB p65/p65	6	24	0.2119	28.31	5.183E-08	12.64
NF-kB2 (p52)	7	44	0.3885	18.02	1.095E-07	10.66

Method: The genes in the “TNFα profile” were used to query to search an interaction database of 22651 genes to ask if the genes in the query are significantly regulated by transcription factors. Significantly over-connected transcription factors have a z-score of >0 with the *p* value dependent on the size of the dataset.

a Actual: number of genes in TNF profile regulated by the chosen TF; R: number of genes in the complete database regulated by the chosen TF. ^b^Expected: mean value for hypergeometric distribution (n*R/N). ^c^Ratio: connectivity ratio (Actual/Expected). ^d^p-value: probability to have the given value of Actual or higher (or lower for negative z-score). ^e^z-score: z-score ((Actual-Expected)/sqrt(variance)).

Going on to examine the effect of SIRT6 and the H133W mutant on gene expression, our first observation was that over expression of SIRT6 without TNFα stimulation (comparison 3) had a small effect on gene expression whereas the over expression of the H133W mutant induced 252 significant gene expression changes (comparison 4).

**Figure 7:**
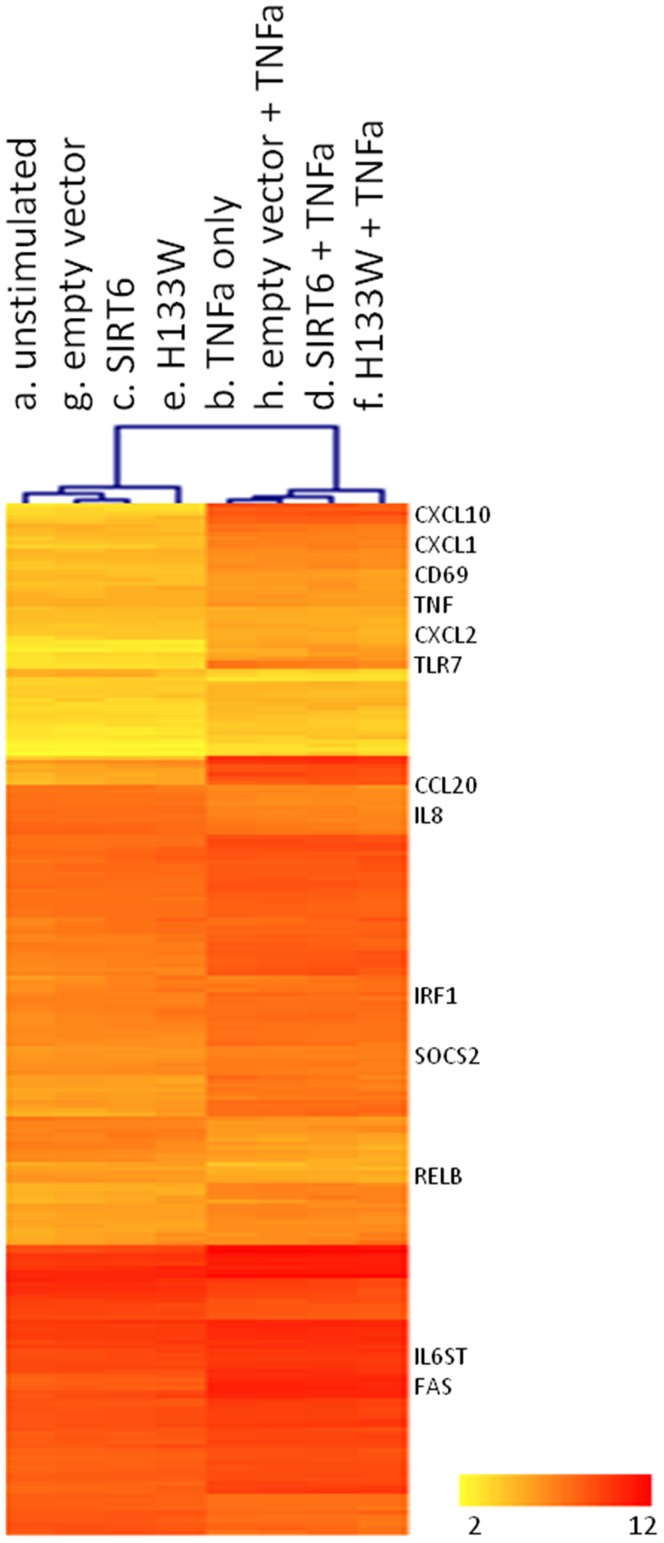
Changes in gene expression of the TNFα profile. Heat map of normalised expression levels (average intensity) of the 198 genes in the TNFα profile under the 8 treatment conditions.

To evaluate the impact of SIRT6 and H133W over expression under TNFα stimulation, we then performed comparisons 5 and 6 in Table 1. We observed that SIRT6 over expression had little effect on the overall profile of TNFα induced gene expression (Figure 7). Similarly, the over expression of the H133W mutant did not have a substantial effect on the “TNFα profile” (Figure 7). Of the top 20 most-changed genes in the “TNFα profile”, only TLR7 is significantly up-regulated in the presence of SIRT6 over expression, and in the presence of the H133W mutant, only CXCL10 was significantly reduced in gene expression. Looking specifically at the effect on MCP1 gene expression in control cells stimulation with TNFα gave a 25.6 fold upregulation of MCP1 mRNA (comparison a vs b, p-value 6.26×10^−11^) whereas in control cells transfected with empty vector MCP1 was upregulated 16.0 fold (comparison g vs h, p-value 4.51×10^−10^). In cells overexpressing SIRT6, TNFα induced a 16.0 fold increase (comparison c vs d, p-value 2.04×10^−10^) and in cells overexpressing H133W there was a 10.2 fold increase (comparison e vs f, p-value 2.57×10^−9^).

From these studies it would appear that SIRT6 does not exert a significant influence on NFκB dependent gene expression under these conditions and one conclusion is that our experimental system is not adequate to reveal a functional response to SIRT6. However, the over expression of H133W resulted in 415 additional genes showing substantial expression changes in comparison to TNFα stimulation alone, with 356 genes being upregulated (comparison 6). The overlap between these 415 gene expression changes in comparison 6 and the 251 gene expression changes in comparison 4 show 168 changes overlap (67%). Pathway enrichment analyses of the 356 genes using a modified version of the PAGE algorithm [24], revealed an excess of genes involved in cell-cycle pathways, specifically the G1-S stage as well as double-stranded DNA damage repair and the NOTCH, WNT and Hedgehog pathways (Table 3). The 356 genes were also found to be over-represented for genes regulated by transcription factors GLI (p value 0.0163; genes NHLH1, GLI1, OLIG2, VEGFA and CD24) and GLI3 (*p* value 0.0207; GLI1). This set of genes was not significantly enriched for NFκB transcription binding sites. The upregulated genes, which included 8 histones, were also highly enriched for genes involved in transcription regulation, cell motility, chromatin assembly and embryonic epithelial cell development [25].

**Table 3:** Pathway enrichment analyses of the 356 genes from comparison 6 using a modified version of the PAGE algorithm.

Pathway	No. of Geneson Pathway	P Value	Pvals_FDR	Genes_on_pathway_and_on_chip	No. of significantgenes on pathway
Cell cycle_G1-S Growth factorregulation	179	6.94E-08	3.30E-05	175	27
DNA damage_Checkpoint	148	4.50E-06	1.47E-03	147	19
Cell cycle_G1-S	206	1.30E-05	3.87E-03	203	25
DNA damage_DBS repair	132	1.71E-05	4.79E-03	124	16
Signal transduction_NOTCHsignaling	225	2.10E-05	5.58E-03	222	29
Signal transduction_WNTsignaling	205	5.83E-05	1.24E-02	199	22
Apoptosis_Apoptoticmitochondria	89	7.17E-05	1.45E-02	87	8
Cell cycle_S phase	177	7.22E-05	1.45E-02	169	20
Cell cycle_G1-S Interleukinregulation	105	1.27E-04	2.25E-02	105	15
Development_Hemopoiesis,Erythropoietin pathway	138	1.42E-04	2.37E-02	135	18
Transcription_mRNA processing	159	1.58E-04	2.52E-02	154	21
Development_Hedgehogsignaling	296	1.82E-04	2.84E-02	291	33
Development_EMT_Regulationof epithelial-to-mesenchymaltransition	272	1.98E-04	2.93E-02	263	28
Cell cycle_Meiosis	159	2.56E-04	3.62E-02	149	19
Cell cycle_G0-G1	89	2.84E-04	3.87E-02	88	15
Cell cycle_G2-M	244	2.88E-04	3.89E-02	237	30

Significant pathways (*p*<0.05) are listed below with the number of genes in the pathway and the number of significant genes in the 356 dataset that are present in the pathway.

## Discussion

The purpose of our study was to develop assays for the discovery of drugs that enhanced the deacetylase activity of SIRT6 and consequently might inhibit NFκB dependent gene expression and act as novel anti-inflammatory or anti-aging medicines. The initial focus of our study was based on recent observations that SIRT6 controls gene expression through its interaction with transcription factors, specifically NFκB and through this mechanism SIRT6 influences inflammation and aging.

We constructed an enzymatic assay for SIRT6 suitable for high throughput drug screening and used this to characterise the kinetics of the enzyme. We showed SIRT6 had a surprisingly low deacetylase activity *in vitro* with values for *k_cat_* of 1.67×10^−5^ s^−1^ and a *K_m, app_* for the peptide substrate of 14 µM thus giving a *k_cat_/K_m_ ratio of* 1.2 M^−1^ s^−1^. Such exceptionally low kinetic values are at the extreme low end in term of enzyme efficiency [26] and raise serious doubts about whether this represents a true enzymatic activity for SIRT6. We are reasonably confident that this does not simply reflect misfolded protein produced in *E. coli* because of the CD spectra and the fact that SIRT6 purified from mammalian cell overexpresion gave similar results. Additionally the *K_m_* for peptide and NAD^+^ appear to be in an acceptable range. We are also confident that the N-terminal tag is not interfering with enzymatic activity since its removal had no influence. To address our doubts about the significance of the deacetylase activity of SIRT6 we over expressed the wild type and mutant forms in mammalian cells and examined the effect on overall levels of H3K9Ac. We confirmed that at the levels of over expression we achieved we were able to substantially reduce or eliminate detectable H3K9Ac in cells with the wild type enzyme whereas the mutants were inactive in this respect although they clearly localised to the cell nucleus. We conclude from these observations that SIRT6 may require another factor or factors to function as an H3K9Ac deacetylase in cells. There could, of course, be catalytic implications on these observations of a low turnover mechanism of SIRT6 deacetylation, or indeed whether this is the primary activity of this enzyme, compared to ADP-ribosylation or even NAD^+^ glycohydrolase activity, as has been reported for PfSir2 [27].

Having established transfection conditions where over expression of SIRT6 could substantially reduce H3K9Ac levels in the nucleus; we sought to explore the consequences of this on NFκB function and NFκB dependent gene expression. We found that over expression of SIRT6 had no detectable influence on the nuclear/cytoplasmic ratio of RelA/p65 nor was the nuclear mobility of RelA/p65 in response to TNFα stimulation affected by over expression of either wild type or H133W SIRT6. If SIRT6 were having an influence on chromatin/DNA association of NFκB we would have expected to see some changes in these parameters, although to be definitive one may have to measure real time oscillations of NFκB in live cells rather than taking a single time point. The significance of this data is that it measures the influence of SIRT6 on a functional readout of NFκB at the single cell level and not an average response in a pool of transiently transfected cells. Over expression of SIRT6 or H133W in HEK cells appeared to have no influence on TNFα induced secretion of MCP1, an inflammatory gene known to be regulated by NFκB, nor did it influence TNFα induced gene expression of MCP1. We went on to perform genome wide transcriptome analysis following TNFα stimulation of HEK293 cells for 4 hours. Our results show a clear signature of TNFα induction of well characterised inflammatory genes, however, over expression of wild type or H133W SIRT6 only had a modest or no significant effect at suppressing this inflammatory transcriptional response.

Thus, by means of a variety of experimental approaches our findings put into doubt whether SIRT6 was exerting a significant influence on NFκB function. A trivial explanation for this lack of effect is that in our transient transfections we do not have sufficient over expression of SIRT6 to show a detectable influence on NFκB. We believe this is unlikely because our transfection efficiency is routinely between 20–50% and we titrated our transfection conditions based on our analysis of the suppression of nuclear H3K9Ac levels (Figure 3). Finally as a positive control, the over expression of H133W did have a profound effect on gene expression. Over expression of this catalytically dead mutant probably exerted a dominant negative effect, suppressing histone deacetylation so maintaining or activating expression of a number of genes. These gene changes add to the body of evidence that SIRT6 does play a role in regulation of transcription, however, in our hands these gene expression changes are not dependent on NFκB. Instead, we found that a key functional component of SIRT6 regulated genes is the Gli transcription factor which controls genes in developmental pathways such as Hedgehog, NOTCH and WNT signalling. Our findings are clearly different from previous results on the transcriptional role of SIRT6 obtained by performing knock outs or knock downs [28]. As an alternative to using siRNA to knock out SIRT6, we chose to use a catalytically dead mutant reasoning that if SIRT6 functions in a multiprotein complex, the function and possibly assembly of that complex may be profoundly disrupted by a knock out. At this stage we have no good explanation why over expression of wild type or dominant negative SIRT6 and knock outs should generate such divergent conclusions. SIRT6 clearly physically interacts with RelA/p65 (as does the H133W mutant, data not shown) and in SIRT6 knock outs this interaction would be lost which may have consequences on NFkB dependent gene expression. Clearly understanding this discrepancy is crucial if we are to understand how a pharmacological inhibitor or activator of SIRT6 would behave.

## Materials and Methods

### Materials

NAD^+^, nicotinamide (NAM) and DTT were purchased from Sigma. Antibodies were purchased as indicated. TNFα was purchased from R&D Systems. Unless otherwise indicated, other chemicals were sourced through Fischer.

### SIRT6 Cloning

Human SIRT6 cDNA was amplified by PCR and cloned into a modified pET28a expression vector (Novagen). This vector was adapted to incorporate a Flag tag upstream of a 6xHIS tag and thrombin (th) cleavage site (CTGGTGCCGCGTGGTAGC LVPRGS). In addition to this Flag-6His-th-tag, the N-terminus of SIRT6 was modified with a Tev (GAAAACCTGTATTTTCAGGGC ENLYFQG) or Precission protease site. Site directed mutagenesis was performed using the Agilent QuikChange II XL Site-Directed Mutagenesis Kit. All constructs were confirmed by DNA sequencing.

### SIRT6 Expression

SIRT6 was expressed in overnight cultures of *E. coli* Rosetta2 DE3 cells in 50×100 ml shake flasks. The total cell pellet mass in these conditions (typically 60 g) was resuspended into 400 mL of lysis buffer (50 mM TRIS HCl, 100 mM NaCl, 20 mM Imidazole, 10% Glycerol (v/v), 1 mM DTT pH8.0+ Roche Protease inhibitor tablets) and sonicated. The clarified lysate was affinity purified on Ni-NTA beads (Qiagen) and eluted with elution buffer (150 mM Imdazole, 50 mM TRIS HCl pH 8.0, 100 mM NaCl,10% Glycerol (v/v), 1 mM DTT + Roche Protease inhibitor tablets). The elute was separated on a HiLoad Superdex 16/60 Prep-grade column equilibrated with 50 mM TRIS HCl pH 8.0, 1 mM DTT, 100 mM NaCl, 10% Glycerol (v/v). To produce SIRT6 without an N-terminal tag, a cDNA construct was made with a Prescission Protease site 3′ to the Flag-6His tag. 15 µL of PreScission Protease (GE Healthcare Bio-Science 27-0843-01) was added to 1.47 mg of tagged-SIRT6 and left on a roller overnight at 4°C. Uncleaved SIRT6 was removed with a Ni-NTA resin and the cleaved SIRT6 was further purified on a HiLoad Superdex 200 10/30 size exclusion column run at 0.5 mL/min in 50 mM TRIS HCl pH 8.0, 1 mM DTT, 100 mM NaCl, 10% Glycerol (v/v). All purified proteins were confirmed by N-terminal Edman sequencing. Protein concentration was determined by the Bradford method.

### Kinetic Characterisation Using an *in vitro* SIRT6 Deacetylase Assay

All peptides were purchased from Cambridge Research Biochemicals. Reactions were performed at 37°C in a 384-well polypropylene plate in buffer containing 50 mM TRIS-HCl pH 7.5, 137 mM NaCl, 2.7 mM KCl, 1 mM MgCl_2_, 1 mM DTT and 0.05% pluronic F-127 (v/v). SIRT6 (1–2 µM final concentration, or higher for the catalytically-compromised mutants) was incubated with 500 µM NAD^+^ and the reaction initiated with a peptide based around the acetylated lysine9 of histone H3: ART(K(QSY7))QTAR(K-acetyl)STGG(K(Tamra))APR (10 µM final concentration). At selected time points, aliquots were removed and added to a Greiner low volume 384-well black assay plate containing nicotinamide (NAM, 40 mM final concentration) to terminate the deacetylation reaction. Following sample collection, endoproteinase Lys-C (9.3×10^−4^ au/ml final concentration, Sigma) was added to cleave the deacetylated SIRT6 peptide and fluorescence was monitored 90 minutes post termination and cleavage, using a Tecan Ultra kinetic microplate reader (excitation 530 nm, emission 590 nm). The rate of SIRT6 deacetylation was calculated from the slope of a linear regression of the individual time-course data under a timeframe where steady-state initial velocity was maintained. The amount of product was quantified in comparison to calibration with known amounts of STGG(K(Tamra))APR peptide. For determination of the apparent *K_m_* values for the peptide substrate, the method was as described above, except that the concentration of peptide was varied from 160 µM whilst the concentration of NAD^+^ was kept constant at 500 µM. Progress curves ascertained at various peptide concentrations over a period of 1 hour. Rates of peptide deacetylation were ascertained using linear regression of the timecourse data over appropriate periods of linearity. Data were fitted to the Michaelis-Menten equation using the Grafit data analysis package.
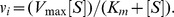



For determination of the apparent *K_m_* values for the NAD^+^ substrate, the method was as described above, except that the concentration of NAD^+^ was varied from 1 mM whilst the concentration of peptide was kept constant at 10 µM. Data were fitted to the Michaelis-Menten equation using the Grafit data analysis package.

### Histone H3/H4 Deacetylation Assay

Histone H3/H4 was purified from HEK293 cells as previously described [29]. 10 µM of purified Histone H3/H4 was incubated with different amounts of SIRT6 for 2 hours at 37°C in deacetylation buffer (10 mM TRIS pH 8.0, 250 mM NaCl, 10% glycerol (v/v), 2.7 mM KCl) in the presence of DTT (0.5 mM) and NAD^+^ (5 mM). The reaction was stopped by addition of 4X NuPAGE sample buffer and heating at 80°C for 10 minutes. Samples were analysed by SDS-PAGE followed by Western blotting and immunodetection with anti-H3K9Ac antibody.

### Cell Transfection and Functional Assays

HEK293 (obtained from ATCC as CRL-1573) cells were routinely transfected with wild type SIRT6 cDNA or mutant forms of SIRT6 cloned into a pcDNA3 expression vector using OptiMEM medium (Invitrogen) and FuGene HD transfection reagent (Roche). 20–50% transfection efficiencies were routinely obtained without obvious cytotoxicity. Cells were transfected with increasing doses of SIRT6 expression vectors (0.5 µg–20 µg/10000 cells per well in a white flat clear bottom 384-well plate). TNFα was added to a final concentration of 10 ng/ml and cells incubated for 48 hours at 37°C. Parallel wells were analysed for cell viability using the Cell Titre Glo assay (Promega) and TNFα-induced MCP-1 secretion into the culture supernatant was normalised to the number of viable cells per well. MCP-1 levels were determined using the MCP-1 Alpha Lisa Kit, (Perkin Elmer). For determination of cellular H3K9 deacetylase activity, HEK293 cells were transfected with SIRT6 or mutant SIRT6 expression vectors as described above. Cells were pre-incubated for 24 hours with the HDAC inhibitor SAHA (1 µM) to increase background levels of histone acetylation. Cells (50 µl at 2×10^5^ cells/ml) were plated out in black clear bottom Poly-D-lysine-coated 384-well plates (Greiner Bio-one, Cell Coat) and allowed to adhere overnight. Cells were fixed with 20 µl/well of 4% paraformaldehyde (v/v) for 10 minutes followed by permeabilisation for 10 minutes with 20 µl of 0.2% TritonX100 (v/v) in PBS followed by addition of 20 µl 1% BSA (w/v), 0.1% Tween 20 (v/v) in PBS for 30 minutes shaking at room temperature. Staining was performed by adding 20 µl of primary antibody and incubating for one hour at room temperature with gentle shaking (1∶1000 anti-H3K9Ac (Millipore 06-942) or 1∶2000 anti-Flag (Sigma) in 1% BSA (w/v), 0.1% Tween (v/v) in PBS). Cells were washed 3 times with PBS, 0.1% Tween (v/v) followed by incubation with fluorescent secondary antibodies diluted 1∶1000. Nuclei were stained with Hoechst dye followed by analysis on a Cellomics array scanner.

### Western Blotting

Transfected cells were lysed on ice for 1 hour in 420 mM NaCl, 20 mM TRIS-HCl pH 7.4, 10 mM KCl, 10 mM MgCl_2_, 2 mM EDTA, 10% glycerol (v/v), 1% Triton X-100 (v/v), protease inhibitor cocktail, followed by gentle sonication. Cell lysates were clarified by centrifugation at 13000 rpm at 4°C for 30 minutes. For western blotting, samples were analysed by SDS-PAGE (NuPAGE, Invitrogen) and transferred to PVDF membrane. Proteins were detected by either anti-FLAG-M2 HRP (Sigma A8592) or with anti-SIRT6 (Sigma S2197 or Bethyl Lab. A302-452A) followed by appropriate HRP-coupled secondary antibodies and ECL reagent (Pierce).

### Immunocytochemistry for RelA/p65 and SIRT6

HEK293 cells were transfected with wild type SIRT6 or SIRT6 mutants as described previously. Following any experimental manipulation, cell were fixed in phosphate-buffered 4% paraformaldehyde (v/v) solution, washed with phosphate-buffered saline (PBS) and permeabilised by 0.2% Triton X-100 (v/v). Autofluorescence was reduced by incubating cells in 15 mM Glycine in PBS, and specific immunofluorescence was enhanced by a further incubation in Image-IT signal enhancer (Invitrogen, Carlsbad, CA, USA). Antibody incubations were carried out in PBS containing 1% Gelatin (w/v) and 0.2% Saponin (w/v) (PGS). Cells were incubated in a mixture of the following primary antibodies for 1 hour: rabbit anti-human NFκB/p65 (Thermo Fisher Scientific, Runcorn, UK) at 20 µg/ml, rat anti-DYKDDDDK Tag (BioLegend, San Diego, CA, USA) at 1 µg/ml and 1/500 monoclonal mouse anti-human SIRT6 (#H00051548-M01, Abnova, Taipei City, Taiwan). Samples were washed in PBS and then incubated in a mixture of goat anti-rabbit Alexa Fluor 488, goat anti-rat Alexa Fluor 568, and goat anti-mouse Alexa Fluor 633 (Invitrogen, Carlsbad, CA, USA) all at 20 µg/ml for 1 hour. After washing, nuclei were counterstained with Hoechst 33342 (Invitrogen, Carlsbad, CA, USA) at 5 µM in PBS for 10minutes at 37°C. Samples were washed and Citifluor anti-fade mountant was added (Agar Scientific, UK), and plates stored at 4°C in the dark before confocal microscopy.

#### Confocal microscopy of immunocytochemistry samples

Confocal microscopy was carried out with a Leica SP5 confocal microscope with Leica LAS software (Leica Microsystems, Milton Keynes, UK). Images were captured with x20 and x63 objective lenses. Sequential images were taken of Hoechst 33342 fluorescence at 405 nm excitation, 450 nm emission, Alexa Fluor 488 at 488 nm excitation, 530 nm emission, Alexa Fluor 568 at 551 nm excitation 600 nm emission, and Alexa Fluor 633 at 633 nm excitation, 670 nm emission. The detector gains were adjusted to avoid saturation of the images that showed the brightest fluorescence, and then the same gain settings were used throughout the experiment. The images were stored as monochrome 8 bit TIF files with 512×512 pixels.

#### Image analysis of immunocytochemistry samples

Image analysis was performed with a Leica Q500 IW image analyser (Leica Microsystems, Milton Keynes, UK) using a customised macro program. The macro performed the following functions: Load monochrome images, combine them into a colour image, allow analysis regions to be defined, detect the immunofluorescence and nuclear stain from background by grey thresholding, and measure the mean intensities of immunostaining in the nucleus and cytoplasm of each cell. The same detection settings were used throughout the experiment. Ratios of nuclear divided by cytoplasmic intensity were calculated and the numbers of positively stained nuclei with intensities >1.25× cytoplasmic intensity were identified.

#### Confocal microscopy of fluorescence recovery of YFP-tagged p65 after photobleaching (FRAP)

This was carried out as previously described [21] using the Leica SP5 confocal microscope and its FRAP software (Leica Microsystems, Milton Keynes, UK), with a x63 water immersion objective lens. Cells with low to moderate nuclear expression of YFP tagged p65 were selected for measurement. The microscope was zoomed in x5 to image single nuclei and rectangular bleach regions of about 0.25 of nuclear area were selected. Images were recorded in a time-lapse sequence once every 270 mseconds. YFP was imaged using 514 nm excitation and 530 nm emission. Two pre-bleach images were taken, followed by 2 bleach frames at maximum 514 nm laser power. Imaging was continued for a further 30 seconds to record the recovery from photobleaching. The Leica software then measured the intensity of YFP fluorescence during recovery within the bleached area and fitted an exponential curve to the data. Twenty-four nuclei for each condition were tested (+/− SIRT6, +/− TNFα) and data transferred to Microsoft Excel for statistical analysis. The half-time to recovery and immobile fraction were recorded and analysed. After FRAP imaging, cells were fixed and immunostained for p65 and SIRT6 as shown above in order to image both YFP-tagged p65 and SIRT6 and the percentages of cells dual transfected was calculated.

### Microarray Gene Expression Analysis

HEK293 cells were plated at 0.4×10^6^ cells per well in 6-well plates (Costar). Cells were transfected with pCDNA3-Flag-SIRT6 WT, pCDNA3-Flag-SIRT6 H133W mutant, pCDNA3 alone or no DNA. Transfection complexes of DNA and FugeneHD™ were formed in OPTI-MEM serum-free media. Cells were grown in media containing transfection complexes for 24 hours prior to stimulation. Growth media was removed and replaced with fresh media containing TNFα (10 ng/ml) or media containing equivalent volume of PBS/0.1% BSA (w/v) as a control and incubated for 4 hours. For each condition, assays were performed in triplicate. There were 8 test conditions examined in this study as follows:

- unstimulated cells;
- TNFα stimulated cells;
- SIRT6 transfected, unstimulated;
- SIRT6 transfected and stimulated with TNFα;
- H133W SIRT6 transfected, unstimulated;
- H133W SIRT6 transfected and stimulated with TNFα;
- empty vector transfected, unstimulated;
- empty vector transfected and stimulated with TNFα.

At 4 hour post stimulation, media was aspirated completely and cells lysed in 300 µL of RLT Plus buffer (Qiagen). Lysates were stored at −80°C until used for RNA extraction (AROS AB, Denmark). Total cellular RNA was isolated, processed, and hybridized to the oligonucleotide microarray HG 133A Plus 2.0 Gene Chip arrays (Affymetrix, Santa Clara, CA) according to the manufacturer’s instructions.

### Analysis of Gene Expression Changes

Fluorescence raw data of the hybridization were computed, RMA normalized, log2 transformed and statistically analyzed using ArrayStudio v4.0 (Omicsoft). Up- and down-regulated genes were defined by the General Linear Model analysis with false discovery rate (FDR)  = 0.05. *P* values were adjusted for multiple testing using Benjamini Hochberg FDR analysis [30]. The complete datasets are deposited in the Gene Expression Omnibus database [series ID GSE28548]. Comparing TNFα-stimulated cells with their unstimulated controls, we considered genes with a fold-change (FC) of >1.5 or < –1.5 and a *p* value of <0.05 as differentially expressed.

## Supporting Information

File S1 **Complete and detailed lists of significant gene expression changes from all comparisons are provided in File S1.**
(XLSX)
