# Correction: Over Expression of Wild Type or a Catalytically Dead Mutant of SIRTUIN 6 Does Not Influence NFκB Responses

**DOI:** 10.1371/annotation/9c1a5765-6471-401f-bc74-a916c8ead46f

**Published:** 2012-09-19

**Authors:** Rachel Grimley, Oxana Polyakova, Jessica Vamathevan, Joanne McKenary, Brian Hayes, Champa Patel, Janet Smith, Angela Bridges, Andrew Fosberry, Anshu Bhardwaja, Bernadette Mouzon, Chun-Wa Chung, Nathalie Barrett, Nicola Richmond, Sundip Modha, Roberto Solari

On page 7 of the PDF there is a large section of text missing from the Discussion. The correct text can be seen in the online version of the manuscript. 

